# An astrocytic cellular model of Lafora disease to study polyglucosan accumulation and inflammation

**DOI:** 10.1242/dmm.052672

**Published:** 2026-02-02

**Authors:** Mireia Moreno-Estellés, Angela Campos-Rodríguez, Rosa Viana, Laura Baños-Carrión, Marta Albuixech, Maria A. García-Gimeno, Matthew S. Gentry, Pascual Sanz

**Affiliations:** ^1^ Instituto de Biomedicina de Valencia, Nutrient Signaling Unit, CSIC, Jaime Roig 11, 46010 Valencia, Spain; ^2^ Department of Biotechnology, Universitat Politécnica de València, 46022 Valencia, Spain; ^3^ Department of Biochemistry and Molecular Biology, University of Florida College of Medicine, Gainesville, FL 32610, USA; ^4^ U742, Centro de Investigación Biomédica en Red de Enfermedades Raras (CIBERER)-Instituto de Salud Carlos III, 46010 Valencia, Spain

**Keywords:** Lafora disease, Astrocyte model, Polyglucosan, Inflammation

## Abstract

Lafora disease (LD) is a devastating form of progressive myoclonus epilepsy characterized by the accumulation of insoluble forms of glycogen [polyglucosan bodies (PGBs)] in the brain and peripheral tissues. It has been proposed that the accumulation of PGBs is pathogenic. Several mouse models of LD have been generated to study the relationship between PGBs and the pathophysiology of LD. However, the use of LD mice is difficult and time consuming; thus, more amenable cellular systems would be desirable. We recently described a cellular model based on the culture of primary postnatal astrocytes from LD mice that are able to accumulate small PGBs. In this study, we extended this astrocytic model by maturing the astrocytes for longer times. These more mature astrocyte cultures accumulated larger and granular PGBs, which have similar properties to the ones present in the hippocampus of *Nhlrc1^−/−^* (*Epm2b^−/−^*) mice. Importantly, this model expresses inflammatory mediators related to LD pathophysiology. This astrocytic model could be used to better understand the formation of the PGBs and also to define how the accumulation of PGBs activates the expression of inflammatory mediators.

## Teaser

**Summary:** An astrocytic cellular model of Lafora disease (LD) accumulates large polyglucosan bodies similar to the ones present in the hippocampus of *Nhlrc1^−/−^* (*Epm2b^−/−^*) mice. This model also expresses inflammatory mediators related to LD pathophysiology.

## INTRODUCTION

Lafora progressive myoclonus epilepsy, also referred to as Lafora disease [LD; Online Mendelian Inheritance in Man (OMIM) #254780, ORPHA501], is a rare, devastating neurological disorder characterized by the accumulation of insoluble forms of glycogen, named Lafora bodies (LBs), in the brain and peripheral tissues (Lafora and Glueck, 1911; Sakai et al., 1970). Affected individuals present with first symptoms during adolescence, with a marked change in behavior, depression and dysarthria. The disease becomes more severe with time, with the appearance of myoclonic episodes, seizures and rapid progressive neurodegeneration, leading to the death of the patient ∼10 years after the onset (Turnbull et al., 2012, 2016; Pondrelli et al., 2021). To date, there is no effective therapy. Patients are initially treated with anti-seizure medications, which, after some time, develop resistance. LD is an autosomal recessive disorder due to mutations in the *EPM2A* gene encoding the glucan phosphatase laforin, or in the *NHLRC1* (also known as *EPM2B*) gene encoding the E3-ubiquitin ligase malin. Laforin and malin form a functional complex that negatively regulates glycogen synthesis. In the presence of a dysfunctional laforin or malin, glycogen synthesis is enhanced, leading to the formation of LBs, which are polyglucosan aggregates with less-branched chains [polyglucosan bodies (PGBs)] (Sullivan et al., 2017). Some years ago, two types of LBs were defined in samples from models of LD: type I, which were polymorphic, granular and dust-like structures, and type II, which were more compacted structures with a dense nucleus (Machado-Salas et al., 2012). Recently, two types of LBs have also been described: small granular PGBs, which are present in cells with a glycolytic metabolism, and large dense PGBs, which accumulate in cells with an oxidative metabolism; the authors suggested that in samples from a patient with LD, neurons accumulate the large dense PGBs, whereas astrocytes accumulate the small granular PGBs (Mitra et al., 2024). This observation is in agreement with a previous report that indicated that the brains of mouse models of LD contain two different types of PGBs: ones that are granular and polymorphic, resembling corpora amylacea, which are present in astrocytes, and others that are more dense and present in neurons (neuronal LBs) (Auge et al., 2018). Interestingly, astrocytes contain most of the PGBs present in the brain of LD mice (Auge et al., 2018; Rubio-Villena et al., 2018).

It has been proposed that the accumulation of PGBs is pathogenic (reviewed in Markussen et al., 2024; Colpaert et al., 2024). However, so far, the molecular basis of the formation of these PGBs and the mechanism by which they trigger the pathophysiology of LD are far from understood. In order to understand the role of PGBs in LD, more amenable cellular systems need to be developed. Cellular models like HEK293 and Neuro2a have been used to study glycogen accumulation (Singh et al., 2012, 2013; Onkar et al., 2025). However, these models are distant from astrocyte physiology. We have recently described that primary astrocytes from LD mice matured with dibutyryl-cAMP (dBcAMP) for 2 weeks accumulate more PGBs than do primary astrocytes from controls (Moreno-Estelles et al., 2023), and we indicated that the possible cause for the accumulation of PGBs was enhanced glucose uptake in the LD astrocyte model in comparison to that in controls.

Here, we present a refined astrocyte model in which primary LD astrocytes are matured for 4 weeks. Under these conditions, cultures accumulate large, granular polyglucosan bodies with properties similar to those in the hippocampus of *Nhlrc1^−/−^* (*Epm2b^−/−^*) mice (from now on, *Epm2b^−/−^* mice), and they upregulate inflammatory mediators implicated in LD pathophysiology. This model enables mechanistic studies of PGB formation and the link between PGB burden and glial inflammatory signaling.

## RESULTS

### Astrocytes from *Epm2b^−/−^* mice accumulate large polyglucosan inclusions after 4 weeks of maturating conditions

**Fig. 1.**
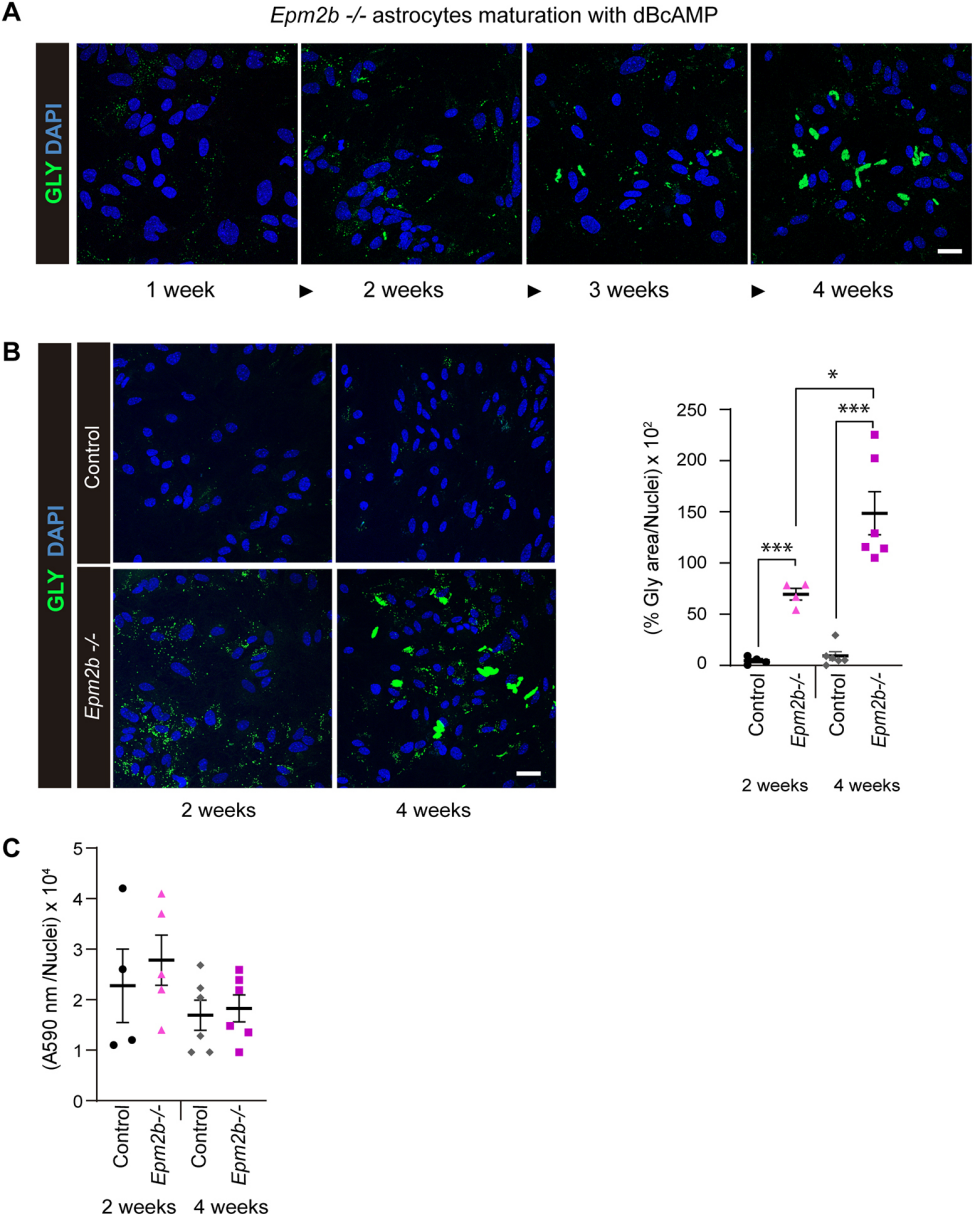
**Large polyglucosan bodies (PGBs) are formed in long-matured primary astrocytes from *Epm2b^−/−^* mice.** (A) Primary cultures of postnatal astrocytes from *Epm2b^−/−^* mice were obtained as described in the Materials and Methods. They were matured with dibutyryl-cAMP (dBcAMP) for different periods, from 1 to 4 weeks. Samples were then analyzed by immunofluorescence using anti-glycogen (GLY) antibody (in green). Nuclei were stained with DAPI (in blue). (B) Immunofluorescence analyses of control and *Epm2b^−/−^* astrocytes matured for 2 weeks or 4 weeks; glycogen is in green, and nuclei were stained with DAPI (in blue). Images were acquired using a 40× objective. Quantification of the glycogen area related to the number of nuclei in the same area is shown on the right. Values are the mean±s.e.m. of at least four independent experiments (*P<0.05, ***P<0.001; unpaired and non-parametric Mann–Whitney test). (C) Viability of the cells was assessed by MTT assay related to the number of nuclei (see Materials and Methods). Values are the mean±s.e.m. of at least four independent experiments. Scale bars: 20 µm.

We previously described a protocol by which astrocytes from mouse models of LD (*Epm2a^−/−^* and *Epm2b^−/−^* mice) accumulate more polyglucosan inclusions (PGBs) than controls (Moreno-Estelles et al., 2023). The accumulated PGBs were small and sensitive to diastase treatment. In addition, we also demonstrated that treatment of the LD astrocytes with 5-aminoimidazole-4-carboxamide ribonucleoside (AICAR) or metformin was able to induce their degradation (Moreno-Estelles et al., 2023). As these LD astrocytes were obtained after 2 weeks of maturation in the presence of dBcAMP, we wondered whether the astrocytes could accumulate more PGBs at longer times. We isolated astrocytes from postnatal day (P)0 to P1 *Epm2b^−/−^* and control pups as previously described, and matured them from 1 week to 4 weeks. As can be observed in Fig. 1A, the amount of the accumulated PGBs increased with the time of maturation in the *Epm2b^−/−^* astrocytes. Astrocytes from control mice did not show a significant accumulation of PGBs with time (Fig. 1B). We quantified the area related to the PGBs at short (2 weeks) and long (4 weeks) maturation conditions. *Epm2b^−/−^* astrocytes displayed a significant increase at 2 weeks (*P*<0.001), which further progressed after 4 weeks (*P*<0.001), compared to controls (Fig. 1B). The glycogen area also increased significantly in the *Epm2b^−/−^* astrocytes after 4 weeks in comparison to 2 weeks of maturation (*P*<0.05). This increase in the area occupied by PGBs was due to the fact that the size of the PGBs accumulated under long maturation conditions was much larger than that of those accumulated under short maturation conditions, although small PGBs were also observed under both conditions (Fig. 1B).

To assess whether the accumulation of PGBs was toxic to the cells, we measured their metabolic activity by assessing the reduction of 3-(4,5-dimethylthiazol-2-yl)-2,5-diphenyltetrazolium bromide (MTT) to purple formazan. As shown in Fig. 1C, astrocytes from *Epm2b^−/−^* mice showed similar general metabolic activity to controls, both at short and long times of maturation, indicating that the accumulation of either small or large PGBs was not toxic to the cells.

### The large PGBs accumulated under long maturation conditions are resistant to diastase and AICAR treatment

**Fig. 2.**
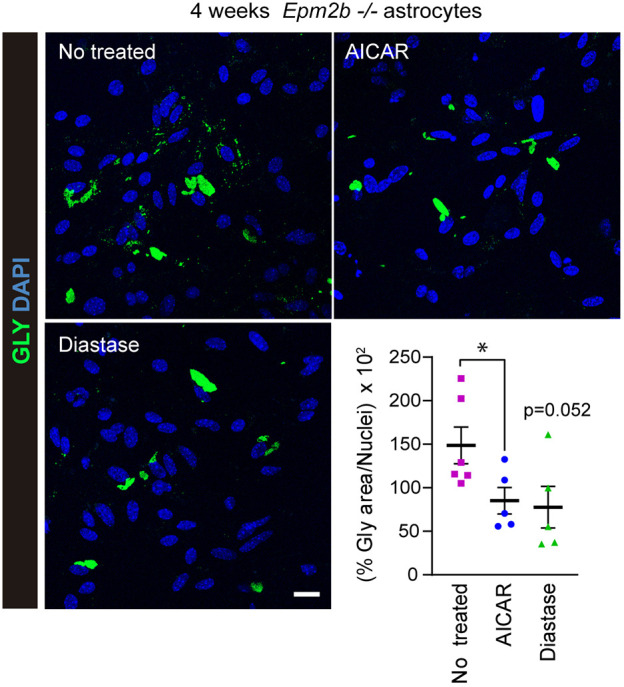
**Sensitivity of the large PGBs present in long-matured primary astrocytes from *Epm2b^−/−^* mice to diastase and AICAR treatments.** Immunofluorescence analysis of the levels of glycogen (in green) in long-matured *Epm2b^−/−^* astrocytes treated with 1 mg/ml diastase for 10 min or with 2 mM 5-aminoimidazole-4-carboxamide ribonucleoside (AICAR) for 24 h. Nuclei were stained with DAPI (in blue). Images were acquired with a 40× objective. Quantification of the glycogen area related to the number of nuclei in the same area is shown in the bottom right. Values are the mean±s.e.m. of at least four independent experiments (*P<0.05; unpaired and non-parametric Mann–Whitney test). Scale bar: 20 µm.

Next, we analyzed the sensitivity of the large PGBs to diastase and AICAR treatments. We observed a trend toward a reduction in the total glycogen area after diastase treatment and a significant reduction after AICAR treatment (*P*<0.05) (Fig. 2). In our opinion, the glycogen reduction observed in long-matured *Epm2b^−/−^* astrocyte cultures corresponds mainly to the degradation of the small PGBs present, contributing to the degradation of the large PGBs only to a minor extent (Fig. 2). Therefore, the large PGBs accumulated after 4 weeks of maturation are mostly resistant to treatment with diastase and AICAR. Importantly, the properties of small and large PGBs accumulated in *in vitro* cultures of *Epm2b^−/−^* astrocytes resemble those observed in LD mice, in which it has been described that the polyglucosan inclusions found in their brain are sensitive to diastase treatment at younger ages, but become resistant to the action of this treatment when the animals age (Rubio-Villena et al., 2018; Auge et al., 2018).

### PGBs from *in vitro* cultured astrocytes are similar to those accumulated *in vivo*

**Fig. 3.**
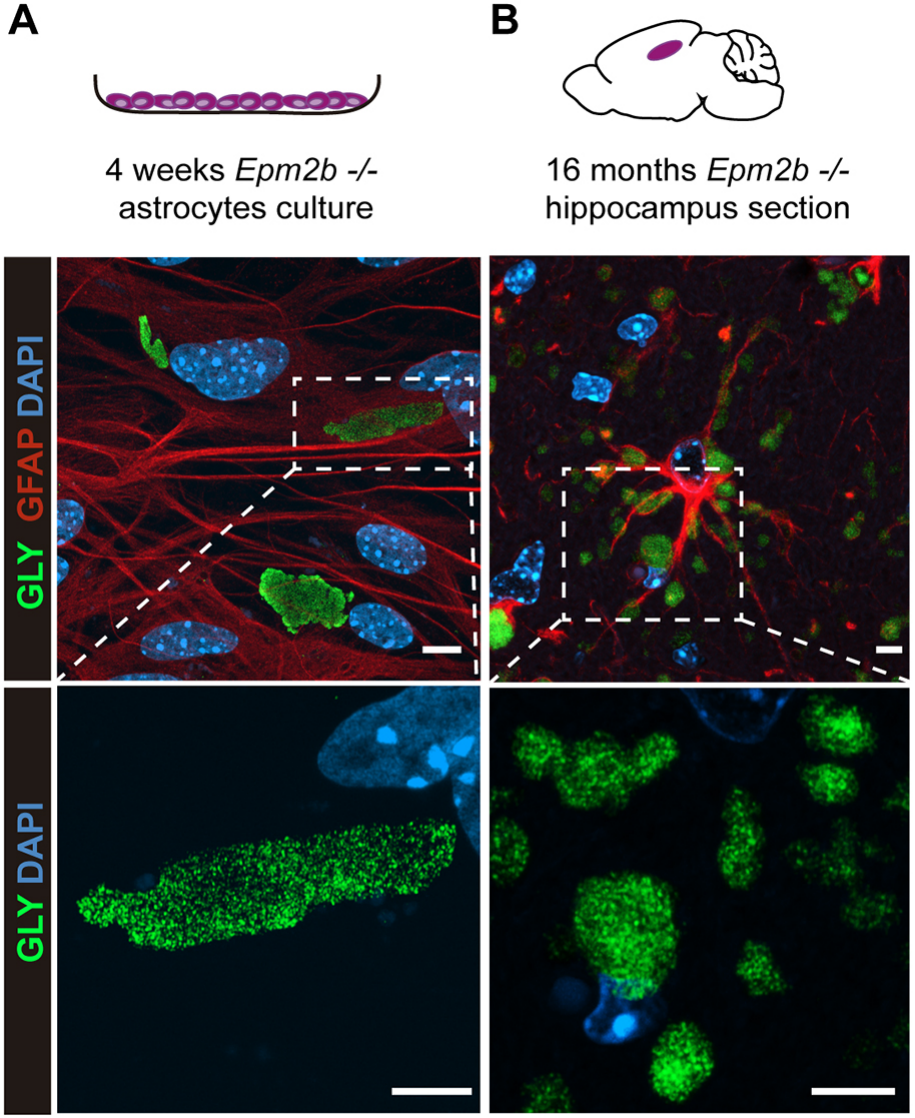
**Microscopy analyses of PGBs present in primary cultures of *Epm2b^−/−^* astrocytes matured for 4 weeks, in comparison to those found in *in vivo* astrocytes from *Epm2b^−/−^* mice of 16 months of age.** (A) Super-resolution microscopy using Airyscan detector of immunofluorescence samples of long-matured *Epm2b^−/−^* astrocytes. Samples were co-stained with anti-GFAP (in red) and anti-glycogen (in green) antibodies; nuclei were stained with DAPI (in blue). Images were acquired using a 63× objective. At the bottom, a magnification of the PGB is shown. (B) Super-resolution microscopy using Airyscan detector of immunofluorescence samples of the hippocampus of *Epm2b^−/−^* mice of 16 months of age. Samples were stained with anti-GFAP (in red) and anti-glycogen (in green) antibodies; nuclei were stained with DAPI (in blue). Images were acquired using a 63× objective. At the bottom, a magnification of the PGBs is shown. Scale bars: 5 µm.

**Fig. 4.**
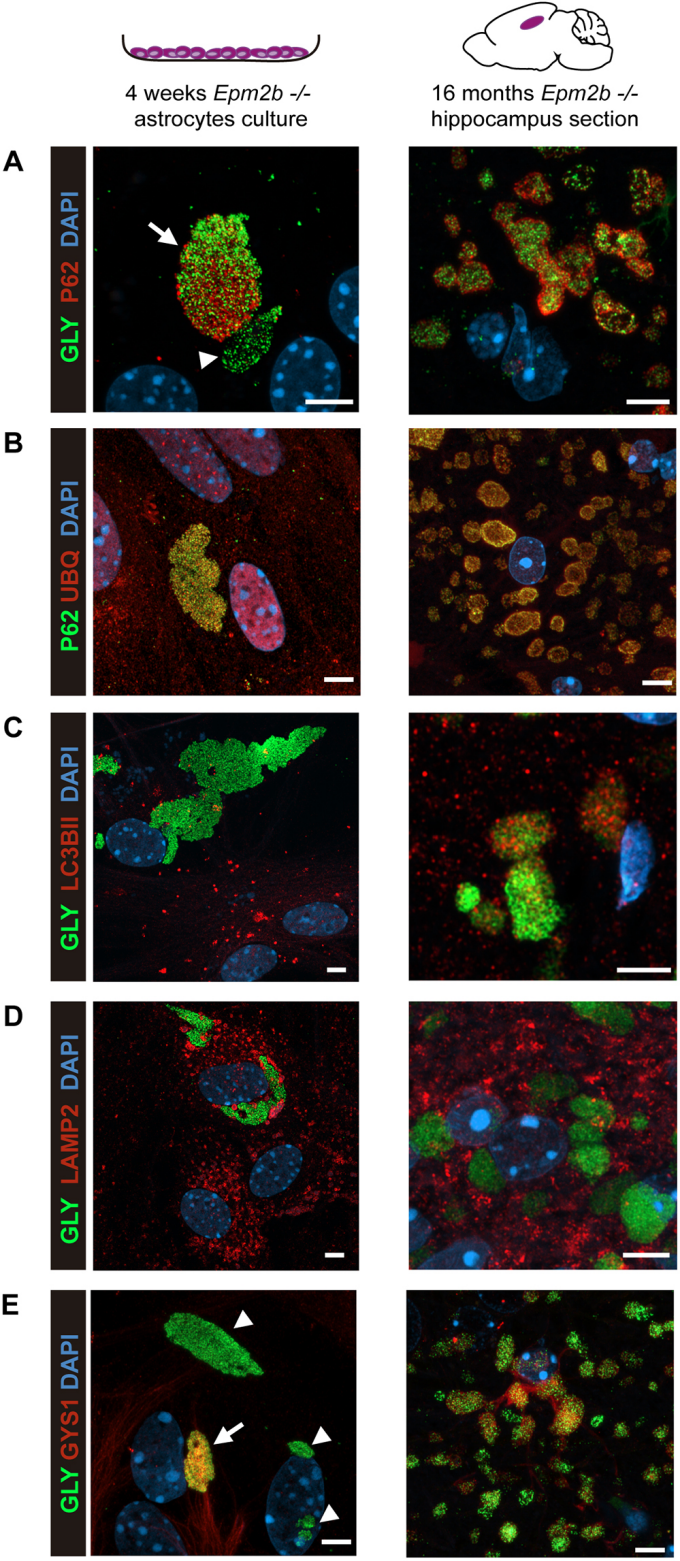
**PGBs from long-matured astrocytes and from *in vivo* astrocytes have similar properties.** (A-E) Super-resolution microscopy using Airyscan detector of immunofluorescence samples of long-matured astrocytes (left column) and the hippocampus of 16-month-old *Epm2b^−/−^* mice (right column), using anti-p62 (in red) and anti-glycogen (in green) antibodies (A); anti-p62 (in green) and anti-ubiquitin (UBQ; in red) antibodies (B); anti-LC3BII (in red) and anti-glycogen (in green) antibodies (C); anti-LAMP2 (in red) and anti-glycogen (in green) antibodies (D); and anti-GYS1 (in red) and anti-glycogen (in green) antibodies (E). Nuclei were stained with DAPI (in blue). Images were acquired using a 63× objective. Arrows indicate PGBs that are positive for the corresponding marker; arrowheads indicate PGBs that are negative for the corresponding marker. Scale bars: 5 µm.

Next, we studied whether the morphology of the PGBs generated in primary cultures of *Epm2b^−/−^* astrocytes is similar to those detected *in vivo*. By using super-resolution microscopy with an Airyscan detector of immunofluorescence samples, we observed that the PGBs present in long-matured astrocyte cultures (*in vitro* PGBs) and those found in the astrocytes of 16-month-old *Epm2b^−/−^* mice at the level of the hippocampus (*in vivo* PGBs), consisted of aggregations of small particles (Fig. 3A,B). Because most of the aggregated materials that accumulate in the cell are targeted for degradation, mainly by autophagy, we analyzed the presence of different markers of autophagy in both PGBs. As observed in Fig. 4A, p62 (SQTSM1), a classical autophagy receptor that binds to polyubiquitinated proteins and targets them to the autophagosome, was present in the large *in vitro* PGBs and partially colocalized with the glycogen particles (arrow in Fig. 4A), although in some PGBs we did not detect p62 (arrowhead in Fig. 4A). In the case of the *in vivo* PGBs, p62 colocalized within the PGBs but was mainly located at the periphery of the particles (Fig. 4A), as already described (Ganesh et al., 2002; Valles-Ortega et al., 2011; Puri et al., 2012; Criado et al., 2012; Pellegrini et al., 2022). We also analyzed the presence of ubiquitinated proteins by using an anti-ubiquitin antibody. We observed similar staining in both types of PGBs (Fig. 4B), which colocalized with the p62 marker. Also, and in agreement with previous results, we observed a surrounding staining of ubiquitin in the *in vivo* PGBs (Criado et al., 2012). Next, we also analyzed the presence of LC3BII (also known as MAP1LC3B), a component of the autophagosomal membrane, and LAMP2, a component of the lysosomal membrane. In the case of LC3BII, we observed partial colocalization of this marker with the PGBs but only in the case of *in vivo* samples (Fig. 4C). By contrast, LAMP2 did not colocalize with the glycogen particles in any of the samples (Fig. 4D). These results are in line with a report that indicates that, in a COS7 cellular model, proteasomal dysfunction leads to accumulated glycogen granules, which are recruited to centrosomal aggresome structures, and that they did not colocalize with autophagosome or lysosome markers (Puri et al., 2011). Finally, we studied the localization of the enzyme responsible for glycogen synthesis in the brain, namely the muscular isoform of glycogen synthase (GYS1) (Vilchez et al., 2007). In this case, in the *in vitro* PGBs, some were GYS1 positive (arrow in Fig. 4E), but others did not stain with this antibody (arrowheads in Fig. 4E). This dual staining was also present in the *in vivo* PGBs (Fig. 4E).

Therefore, despite some differences, the *in vitro* postnatal astrocytes matured for 4 weeks are a good model to study the accumulation of PGBs in *Epm2b^−/−^* mice.

### Induction of pro-inflammatory mediators in long-matured astrocyte cultures

**Fig. 5.**
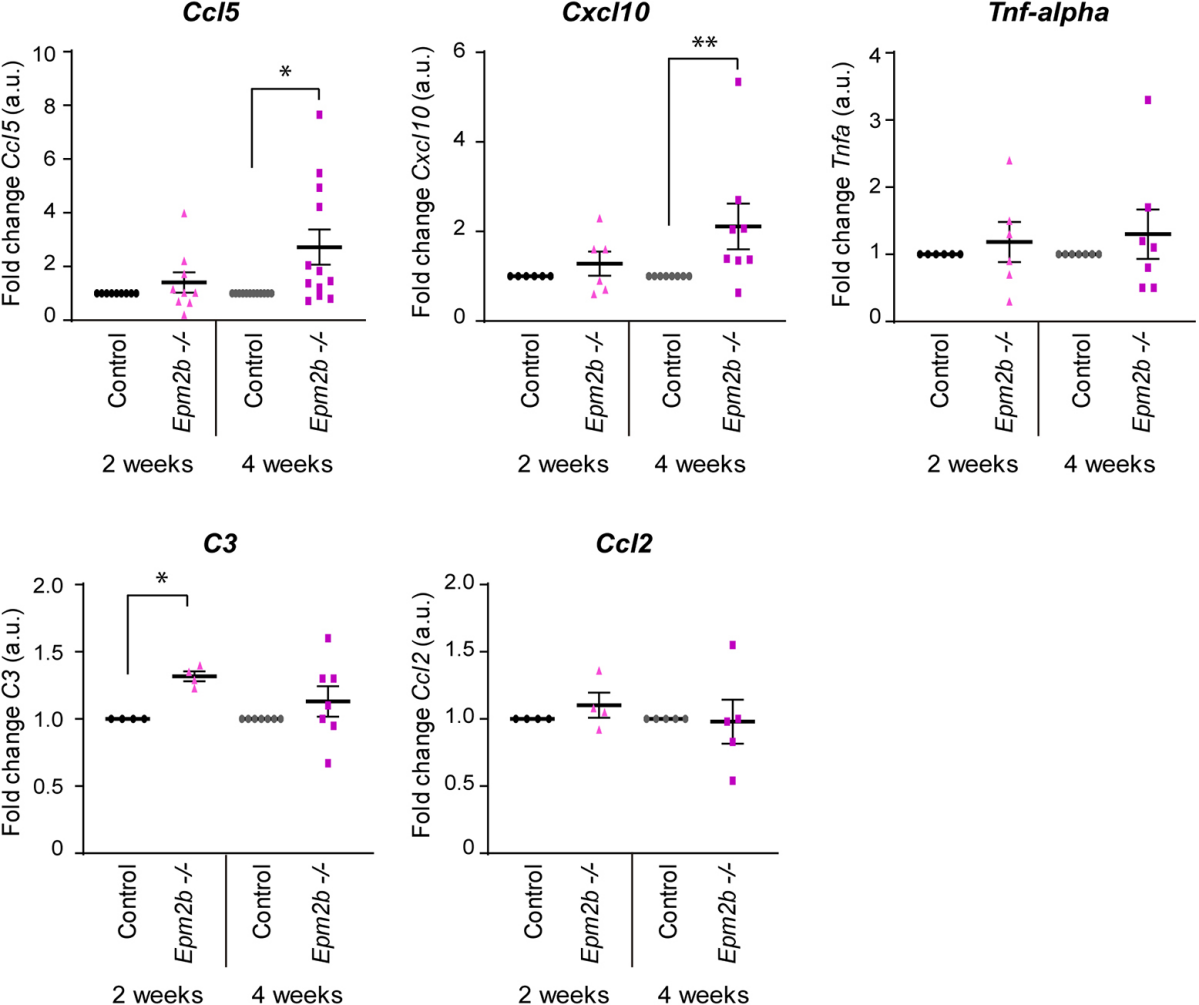
**RT-qPCR analysis of inflammatory mediators in short and long-matured astrocytes.** Relative expression levels of *Ccl5*, *Cxcl10*, *Tnf-alpha*, *C3* and *Ccl2* from short- and long-matured control and *Epm2b^−/−^* astrocytes were analyzed by reverse transcription quantitative PCR (RT-qPCR) in at least four independent samples and three technical replicates. The expression levels of each gene were normalized to those of *Actb*. Values are the mean±s.e.m. of the fold change in comparison to the corresponding control sample. Statistical comparisons were made using an unpaired and non-parametric Mann–Whitney test (*P<0.05, **P<0.01). a.u., arbitrary units.

We recently described that astrocytes play a key role in the development of neuroinflammation in LD through the release of different pro-inflammatory mediators (Lahuerta et al., 2020; Rubio et al., 2023). Although our results indicate that the accumulation of PGBs in cultured astrocytes does not affect viability (Fig. 1C), we checked whether the presence of PGBs could induce the production of different molecules related to neuroinflammation. With this aim, we analyzed the expression of *Ccl5*, *Cxcl10*, *Tnf-alpha* (also known as *Tnf*), *C3* and *Ccl2* pro-inflammatory mediators in astrocytes subjected to short (2 weeks) and long (4 weeks) maturation conditions. As observed in Fig. 5, in short-matured *Epm2b^−/−^* astrocytes, we only found changes in the expression of *C3* (*P*<0.05) among all these markers, in comparison to those in control astrocytes. In the case of long-matured *Epm2b^−/−^* astrocytes, we found a significant increase in the production of the *Ccl5* (*P*<0.05) and *Cxcl10* (*P*<0.01) pro-inflammatory mediators, in comparison to that in the long-matured control astrocytes (Fig. 5). These results suggest that, possibly owing to the presence of PGBs, the *Epm2b^−/−^* astrocytes initiate the production of pro-inflammatory mediators.

## DISCUSSION

LD is a progressive disorder with no treatment available yet. Different strategies have been used to find an appropriate treatment that could cure or at least ameliorate the symptoms of the disease (Markussen et al., 2024; Colpaert et al., 2024). Multiple studies suggest that the accumulation of PGBs present in animal models of LD is pathogenic (reviewed in Markussen et al., 2024; Colpaert et al., 2024). The use of these animal models is resource intensive; therefore, more tractable cellular systems are needed as alternative models. We recently described a protocol by which primary cultures of astrocytes matured for 2 weeks from mouse models of LD (*Epm2a^−/−^* and *Epm2b^−/−^* mice) accumulated more polyglucosan inclusions (PGBs) than controls (Moreno-Estelles et al., 2023). Herein, we present an improved cellular model based on the use of cultures of primary LD astrocytes, which have been matured for 4 weeks. Under these conditions, LD primary astrocytes accumulate large PGBs that resemble the ones that accumulate in the brains of LD mice: (1) they are resistant to digestion with diastase; (2) in both the *in vitro* and *in vivo* systems, astrocytic PGBs are formed by the aggregation of small particles; (3) they are not surrounded by membranes containing either LC3B (autophagosomes) or LAMP2 (lysosomes) proteins; (4) they contain p62, an autophagy receptor that recognizes ubiquitinated proteins, and ubiquitin, although in the *in vivo* PGBs, p62 and ubiquitin present a clear distribution in the periphery of the particle; and (5) they contain GYS1, the enzyme responsible for glycogen synthesis. Therefore, we conclude that the culture of primary astrocytes of LD mice that have matured for at least 4 weeks constitutes a suitable model to study the formation of the PGBs that accumulate in the mouse models of LD.

The fact that the astrocytic PGBs are composed of small aggregates favors the hypothesis that the accumulation of PGBs starts with the formation of small particles (Brewer et al., 2020; Neoh et al., 2024). These particles would associate with each other, forming a high-complexity structure that will start accumulating proteins on the surface, leading to the formation of PGBs (Brewer et al., 2020; Neoh et al., 2024). In agreement with this hypothesis, we show evidence that in the *in vitro* astrocytic PGBs, in which the small aggregates are more loosely bound, the whole structure containing the granules is not surrounded by p62 (Fig. 4A) or ubiquitinated proteins (Fig. 4B). In contrast, in the *in vivo* astrocytes, the signal of p62 (Fig. 4A) and ubiquitinated proteins (Fig. 4B) is mainly located at the periphery of the whole structure containing the granules, possibly because they are more compacted.

Perhaps, the small differences observed between both models are either due to the fact that the *in vitro* astrocyte model lacks other cellular components present in the *in vivo* system (neurons, microglia, etc.), or to the fact that the PGBs in the *in vitro* model come from primary astrocytes from newborn animals matured in the incubator for 4 weeks, whereas the *in vivo* PGBs analyzed by immunofluorescence come from 16-month-old animals.

In any case, our work demonstrates that long-matured LD astrocytes can produce several pro-inflammatory mediators, such as C3, Ccl5 and Cxcl10, which were identified among the different inflammatory markers present in the brains of LD mice (Lahuerta et al., 2020). Because control astrocytes matured under the same conditions did not produce any of these mediators, we suggest that the accumulation of the large PGBs present in the model of LD is the trigger for the production of pro-inflammatory molecules, which could mediate in the activation of the neuroinflammation present in LD mice. This adds extra value to our model of LD as it will allow the study of how the inflammatory reaction is generated and also how it can be prevented. These astrocytes could be also used to screen drug libraries in order to identify compounds that could prevent the accumulation of PGBs or induce their degradation.

Overall, cellular models offer several advantages over *in vivo* animal models. The experimental design is simpler and more reproducible, as greater control can be exerted over the variables being tested, and molecular processes can be studied without interference from other physiological systems. *In vitro* models can also be easily modified (e.g. through transfection or gene editing) to study the functions of specific genes or proteins, and they are cheaper and faster than breeding, maintaining and experimenting with animals. This approach aligns with the 3Rs principles (Replacement, Reduction and Refinement) of biomedical research, as it avoids animal suffering and death. Additionally, this new cellular model for LD enables researchers to focus on the cell population showing the highest glycogen accumulation, the astrocytes, and it recapitulates the formation of large glycogen accumulations and p62- and ubiquitin-positive inclusions in just one month, which are similar to those observed in animal models of LD.

## MATERIALS AND METHODS

### Primary mouse astrocyte isolation and culture

This study was carried out according to the recommendations in the Guide for the Care and Use of Laboratory Animals of the Consejo Superior de Investigaciones Científicas (CSIC; Spain) and approved by the Consellería de Agricultura, Medio Ambiente, Cambio Climático y Desarrollo Rural from the Generalitat Valenciana. Mouse procedures were approved by the animal Ethics committee of the Instituto de Biomedicina de Valencia-CSIC (Permit Number IBV-51, 2019/VSC/PEA/0271). All efforts were made to minimize animal suffering. Mouse primary astrocytes from control and *Epm2b^−/−^* mice were obtained from P0 to P1 mice as in Moreno-Estelles et al. (2023). Cells were grown in Dulbecco's modified Eagle medium (DMEM; Lonza, Barcelona. Spain) containing 20% inactivated fetal bovine serum (FBS; Fisher Scientific, Madrid, Spain), supplemented with 1% L-glutamine, 7.5 mM glucose, 100 units/ml penicillin and 100 μg/ml streptomycin, in a humidified atmosphere at 37°C with 5% CO_2_. After 48 h, FBS was reduced to 10%. For the following 2 (short) to 4 (long) weeks, 0.25 mM dBcAMP (D0627, Sigma-Aldrich, Madrid, Spain) was added to the cultures to favor astrocyte maturation. At the end of the maturation process, primary astrocytes were grown for a further 24 h in the same media but in the absence of dBcAMP to avoid any secondary effect deriving from the compound (Hertz et al., 1998; Muller et al., 2014).

### Immunofluorescence analyses

#### Immunocytofluorescence

Cells plated in a glass coverslip were fixed with 4% paraformaldehyde (PFA) in phosphate-buffered saline (PBS) for 15 min, washed with PBS and immersed for 1 h in blocking buffer 1% bovine serum albumin (BSA), 10% FBS, 0.2% Triton X-100, in PBS; or 3% BSA, 0.02% saponin in PBS in the case of anti-LC3BII and anti-LAMP2 antibodies. Cells were incubated overnight at 4°C with the corresponding primary antibodies diluted in the corresponding blocking buffer (see Table S1). After three 10 min washes in PBS, cells were incubated for 1 h at room temperature with the appropriate secondary antibody diluted 1:500 in blocking buffer, washed twice with PBS and mounted in Fluoroshield with DAPI (F6057, Sigma-Aldrich, Madrid, Spain). When indicated, samples were first treated with 1 mg/ml diastase (alpha-amylase; A3176, Sigma-Aldrich) in PBS for 10 min at 37°C; then, they were washed three times (10 min each) with PBS and processed for immunofluorescence analyses as above. Images were acquired in an LSM 980 confocal microscope (Zeiss, Oberkochen, Germany). *Z*-stack series of 0.5 μm covering the entire cell were acquired using the 63× objective, at one unit Airy pinhole, with a resolution of 1024×1024 pixels. When indicated, images were acquired using a 40× objective, at one unit Airy pinhole, with a resolution of 512×512 pixels and *z*-stacks of 0.8 µm. For each staining, the percentage of the corresponding laser intensity and detector gain settings was maintained between samples. For image analysis, the background obtained using only a secondary antibody was subtracted using the image-processing package FIJI-ImageJ, and the intensity of the signal was normalized by the size of the studied area. When indicated, the Airyscan detector in the super-resolution mode was used. In that case, multiple *z*-stacks with an interval of 0.15 μm were imaged to ensure that the entire cell was captured. Images were directly processed using the Airyscan processing from Zeiss Zen Blue program and presented as the maximum projections of the *z*-stacks.

#### Immunohistofluorescence

Mice aged 16 months were deeply anesthetized with sodium pentobarbital (80 mg/kg) administered by intraperitoneal injection. Cardiac perfusion was then performed using a perfusion pump at a flow rate of 8 ml/min with 4% PFA in PBS, delivering 40-48 ml of fixative per animal. Initially, the circulatory system was flushed with 24 ml of 0.9% saline solution to remove blood before introducing the fixative. After perfusion, brains were removed and incubated 2 h in 15 ml of 4% PFA at 4°C with shaking. Then, they were washed three times with PBS at room temperature for 10 min per wash. Next, brains were incubated for 30 min with 50% ethanol, followed by overnight incubation in 70% ethanol. The next day, the two hemispheres were separated, dehydrated, cleared and embedded in paraffin. The samples embedded in paraffin were sagittally sectioned at 4 μm using a HistoCore Biocut microtome (Leica, Madrid, Spain). Brain sections were deparaffined, rehydrated, and microwave antigen retrieval was performed for 10 min in 10 mM citrate buffer pH 6.0. Sections were immersed in blocking buffer (1% BSA, 10% FBS, 0.2% Triton X-100, in PBS) for 1 h and incubated overnight at 4°C with the corresponding primary antibodies diluted in blocking buffer. After three 10 min washes in PBS, samples were incubated for 1 h at room temperature with the appropriate secondary antibody diluted 1:500 in blocking buffer without Triton X-100, washed once with PBS, incubated with DAPI (Sigma-Aldrich), washed twice with PBS and mounted in AquaPolymount (Polysciences, Warrington, PA, USA). Images were acquired as above.

### MTT determination

Cell culture medium was removed, and cells were incubated with 0.2 mg/ml MTT solution (M5655, Merck, Madrid, Spain) for 1 h at 37°C with 5% CO₂. Following incubation, cells were gently washed twice with 1× PBS and lysed with DMSO. Absorbance at 590 nm was measured in three technical replicates in a Varioskan LUX Multimode Microplate Reader (Thermo Fisher Scientific, Madrid, Spain).

### Reverse transcription quantitative PCR (RT-qPCR) analyses

The expression of *Tnf-alpha*, *Cxcl10*, *Ccl5*, *Ccl2* and *C3* was measured in the samples using SYBR Green-based RT-qPCR as in Lahuerta et al. (2020). In brief, short- and long-matured primary cultures of control and *Epm2b^−/−^* astrocytes were lysed with TriPure Isolation Reagent (11667165001, Merck) and RNA purified as in Rubio et al. (2023). For each reaction, 1 µg total RNA from each sample was reverse transcribed with the PrimeScript™ RT reagent kit (RR037A, Takara, Shiga, Japan) under the following conditions: 37°C for 15 min, 85°C for 5 s, and hold at 4°C. The resulting cDNA was amplified and quantified with TB Green^®^ Premix Ex Taq (RR420A, Takara, Shiga, Japan). The primer sequences were those described in Lahuerta et al. (2020). SYBR Green-based RT-qPCR was performed under the following conditions: 95°C for 10 min, followed by 40 cycles of 95°C for 15 s, 60°C for 1 min, and 60°C to 95°C in increments of 0.5°C for 30 s to generate melting curves. The data were processed using StepOnePlus software version 2.3, and expression values were calculated using the comparative Ct method. Each qPCR reaction was performed on at least four independent biological samples. The β-actin (*Actb*) gene was used as the endogenous reference control to normalize target gene expression.

### Statistical analysis

Results are shown as means±s.e.m. of at least three independent experiments. Statistical comparisons were made using an unpaired and non-parametric Mann–Whitney test using Prism version 5.0 (Graph Pad Software, La Jolla, CA, USA). *P*<0.05 was considered significant.

## Supplementary Material

10.1242/dmm.052672_sup1 Supplementary information
