# Supplementary material for: An astrocytic cellular model of Lafora disease to study polyglucosan accumulation and inflammation
Source: Dis Model Mech. 2026 Feb 2;19(1):dmm052672. doi: 10.1242/dmm.052672 (PMC12919960; doi:10.1242/dmm.052672)
Supplement: Supplementary information [file dmm-19-052672-s1.pdf]

Table S1. Primary antibodies used in immunofluorescence analyses.

**Primary antibody**	**Reference**	**Concentration**
anti-GFAP	173308, Synaptic systems	1:1000
anti-Glycogen	Gifted from Dr Otto Baba; Tokyo Medical and Dental University, Tokyo, Japan	1:500
anti-GYS1	ab40810, Abcam	1:100
anti-LAMP2	PA1-655, Invitrogen	1:1000
anti-LC3BII	PM036, MBL	1:1000
anti-S100B	ab52642, Abcam,	1:500
anti-p62	GP-62-C, Progen	1:600
anti-Ubiquitin FK2	BML-PW8810, Enzo	1:100

Disease Models & Mechanisms • Supplementary information
